# Weekly nanoparticle albumin-bound paclitaxel and paclitaxel for relapsed small cell lung cancer: A retrospective observational study

**DOI:** 10.1097/MD.0000000000028863

**Published:** 2022-02-11

**Authors:** Hajime Oi, Toshiaki Matsuda, Tomoki Kimura, Masahiro Morise, Yasuhiko Yamano, Toshiki Yokoyama, Kensuke Kataoka, Yasuhiro Kondoh

**Affiliations:** a Department of Respiratory Medicine and Allergy, Tosei General Hospital, Seto, Aichi, Japan; b Department of Respiratory Medicine, Nagoya University Graduate School of Medicine, Nagoya, Aichi, Japan.

**Keywords:** nab-paclitaxel, paclitaxel, relapsed, small cell lung cancer

## Abstract

In addition to advanced non-small cell lung cancer, nanoparticle albumin-bound paclitaxel (nab-PTX) may also harbor potential benefit for patients with relapsed small cell lung cancer (SCLC), since weekly paclitaxel (PTX) shows modest activity for relapsed SCLC. We evaluated the efficacy and safety of both weekly nab-PTX and PTX for relapsed SCLC.

We retrospectively reviewed 52 consecutive relapsed SCLC patients who were treated with weekly nab-PTX or PTX at our hospital.

The response rate, median progression-free survival and overall survival with nab-PTX and PTX were 5.6 vs 8.8%, 3.2 vs 1.7 months, and 5.4 vs 4.5 months, respectively. No statistically significant differences were observed. There was no statistical difference between the 2 groups for ≥Grade 3 adverse events.

Weekly nab-PTX and PTX showed similar activity for relapsed SCLC. The toxicity profile of nab-PTX was equally tolerable to that of PTX.

## 1 Introduction

Lung cancer is the leading cause of cancer-related deaths worldwide. Small cell lung cancer (SCLC) accounts for about 14% of all lung cancer.^[1]^

However, almost all extensive-stage patients and 75% of limited-stage patients experience a disease relapse after initial therapy. In the treatment of relapsed SCLC, topotecan and cisplatin plus etoposide with irinotecan are the standard 2^nd^-line options for sensitive relapse patients, defined as those with relapse at an interval of 90 to 180 days after the completion of initial chemotherapy.^[2–4]^ In contrast, treatment options are limited in patients with refractory relapse, defined as no response to initial chemotherapy or relapse within 90 to 180 days after the completion of initial chemotherapy. Based on the data from phase II trials, paclitaxel (PTX), irinotecan, and nivolumab ± ipilimumab (if immuno-checkpoint inhibitor is not used in 1^st^-line treatment) are recommended for 2^nd^-line options in the National Comprehensive Cancer Network Clinical Practice Guidelines. Although the efficacy of these agents is modest, toxicity induced by 2^nd^-line chemotherapy is a clinical issue associated with treatment termination because relapsed SCLC patients often show poor tolerability due to deterioration of performance status. Additionally, some patients have both SCLC and interstitial lung disease (ILD), but irinotecan and immune-checkpoint inhibitors are difficult to use for patients with ILD due to lung toxicity. Therefore, less toxic treatments are sought.

Nanoparticle albumin-bound paclitaxel (nab-PTX), a nanoparticle conjugate of PTX to albumin, is designed to improve PTX solubility. This results in reduced toxicity from PTX, such as infusion reaction. In advanced non-small cell lung cancer (NSCLC), carboplatin plus weekly nab-PTX has been shown to be equal to carboplatin plus PTX combined with carboplatin, with fewer neurological adverse events (AEs).^[5]^ Weekly nab-PTX therapy also shows modest activity, with a 31.7% response rate in patients with pre-treated advanced NSCLC in 1 study.^[6]^ In addition to advanced NSCLC, nab-PTX could harbor potential benefit for patients with relapsed SCLC since weekly PTX shows modest activity for relapsed SCLC.^[7,8]^

Several reports of weekly nab-PTX and weekly PTX treatment for relapsed SCLC have described its efficacy and safety, but they are limited cases and has not been fully discussed.^[7–14]^ Based on such evidence, we conducted the current retrospective study to evaluate the efficacy and safety of both weekly nab-PTX and weekly PTX regimens for relapsed SCLC.

## 2 Patients and methods

### 2.1 Study population

This retrospective cohort study was carried out under the principles of the Declaration of Helsinki and approved by the Tosei General Hospital Institutional Review Board, Seto, Japan (IRB No. 789). We retrospectively reviewed consecutive relapsed SCLC patients who were treated with weekly nab-PTX or weekly PTX at Tosei General Hospital between January 2008 and March 2019.

Patients were selected according to the following criteria: histologically diagnosed as SCLC, Eastern Cooperative Oncology Group Performance Status (ECOG-PS) of 0 to 3, at least 1 measurable lesion defined by Response Evaluation Criteria in Solid Tumors version 1.1.^[15]^ The exclusion criteria of this study were inadequate organ function and ECOG-PS of 4. The aim of this study was to evaluate the efficacy and safety profile of both weekly nab-PTX and weekly PTX. Objective response rate (ORR), disease control rate (DCR), progression-free survival (PFS), and overall survival (OS) were set as efficacy outcomes. All AEs during weekly nab-PTX or weekly PTX therapy were recorded according to Common Terminology Criteria for Adverse Events (version 4.0). The data cut-off date for this analysis was June 30, 2019.

### 2.2 Treatment regimens

Patients were treated with nab-PTX alone or PTX alone. The treatment regimen of weekly nab-PTX was 80 mg/m^2^, days 1, 8, 15/q4 weeks and that of weekly PTX was 80 mg/m^2^, days 1, 8, 15, 22, 29, 36/q8 weeks, until radiographically confirmed disease progression, unacceptable toxicity, withdrawal, or death. Nab-PTX became available for use in Japan on February 21, 2013, and has been used sequentially at our hospital thereafter.

### 2.3 Evaluations of efficacy and toxicity

We analyzed the subjects’ clinical characteristics, treatment courses, the clinical efficacy of weekly nab-PTX or weekly PTX, and AEs. ORR, DCR, PFS, and OS were set as efficacy outcomes. ORR was evaluated using Response Evaluation Criteria in Solid Tumors version 1.1. OS was defined as the time from the first administration of the chemotherapy until the final visit or death. PFS was defined as the time from the first administration of the chemotherapy to the date of confirmation of disease progression or death.

### 2.4 Statistical analysis

Survival curves were prepared using the Kaplan-Meier method and the differences in PFS and OS between weekly nab-PTX and weekly PTX were compared using the log-rank test. Mann-Whitney test and Fisher exact test were used to analyze the difference in the 2 groups. Differences with 2-sided *P* values of <.05 were considered statistically significant. All statistical analysis was performed using the EZR (The R Foundation for Statistical Computing, Vienna, Austria).^[16]^

## 3 Results

### 3.1 Patient characteristics

A total of 52 patients with relapsed SCLC were reviewed; 18 and 34 patients received weekly nab-PTX and weekly PTX, respectively. The patients’ characteristics are shown in Table 1. Median follow up periods were 5.2 months (range, 0.2–14.2 months) with nab-PTX and 4.9 months (range, 0.4–55.6 months) with PTX. Median age was 73 years (range, 54–80 years) with nab-PTX and 70 years (range, 49–81 years) with PTX. The majority of patients had a good ECOG-PS (ECOG-PS 0–1: 18 [100%] with nab-PTX, 29 [85%] with PTX, *P* = .456). The proportion of patients who had undergone previous thoracic radiation therapy, and those with sensitive relapse in the nab-PTX group were also statistically significantly lower than those in the PTX group (sensitive relapse: 2 [11%] with nab-PTX, 14 [41%] with PTX, *P* = .031. Previous thoracic radiation therapy: 1 [6%] with nab-PTX, 10 [29%] with PTX, *P* = .039). The PTX group patients had more previous chemotherapy regimens than the nab-PTX patients (*P* = .031).

**Table 1:** Patient characteristics.

Baseline characteristics		nab-PTX (n = 18)	PTX (n = 34)	*P* value
Sex	Male	17 (94%)	28 (82%)	.399
Age		73 (54–80)	70 (49–81)	.298
Smoking status	Ever/Never	17 (94%)/1 (6%)	33 (97%)/1 (3%)	.999
ECOG-PS	0/1/2/3	3/15/0/0	7/22/4/1	.456
	0–1	18 (100%)	29 (85%)	.15
Disease extent	Limited/Extensive	4 (22%)/14 (78%)	17 (50%)/17 (50%)	.076
Previous thoracic radiation therapy	Yes	1 (6%)	10 (29%)	.039
Previous chemotherapy regimen^∗^	Platinum + etoposide	13 (72%)	30 (88%)	.247
	Platinum + irinotecan	7 (39%)	13 (38%)	.999
	Amrubicin	9 (50%)	19 (56%)	.774
	Irinotecan	0	7 (20%)	.081
No. of previous chemotherapy regimens	1-2/3-	16/2	20/14	.031
First line chemotherapy efficacy	Sensitive/Refractory	2 (11%)/16 (89%)	14 (41%)/20 (59%)	.031
Interstitial lung disease		10 (55%)	16 (47%)	.771
COPD		4 (22%)	7 (20%)	.999

No. or range. COPD = chronic obstructive pulmonary disease, ECOG-PS = Eastern Cooperative Oncology Group Performance Status, nab-PTX = nanoparticle albumin-bound paclitaxel, PTX = paclitaxel.

∗ Overlap.

Respiratory comorbidities included 10 (55%) patients with ILD and 4 (22%) with chronic obstructive pulmonary disease in the nab-PTX group, and 16 (47%) patients with ILD and 7 (21%) with chronic obstructive pulmonary disease in the PTX group.

### 3.2 Efficacy

**Figure 1:**
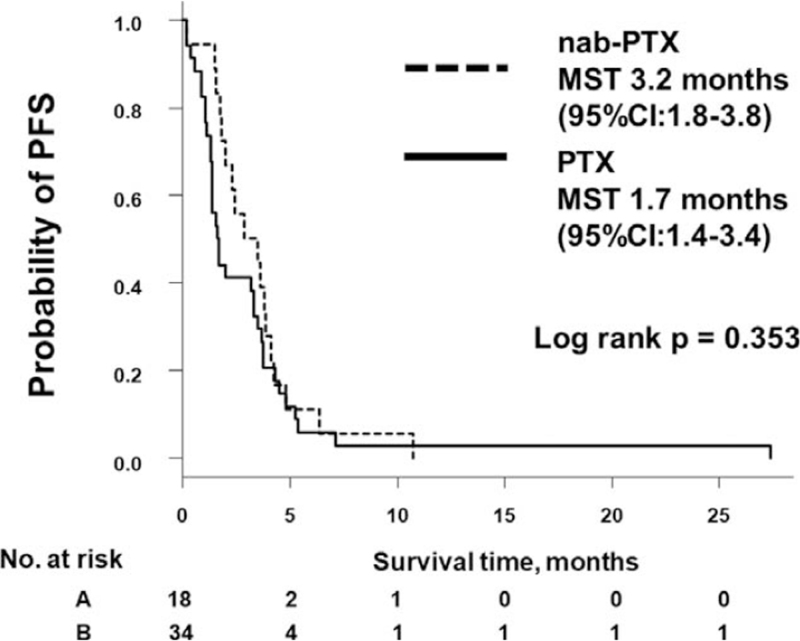
Kaplan-Meier analysis of PFS rate. CI = confidence interval, MST = median survival time, nab-PTX = nanoparticle albumin-bound paclitaxel, PFS = progression-free survival, PTX = paclitaxel.

**Figure 2:**
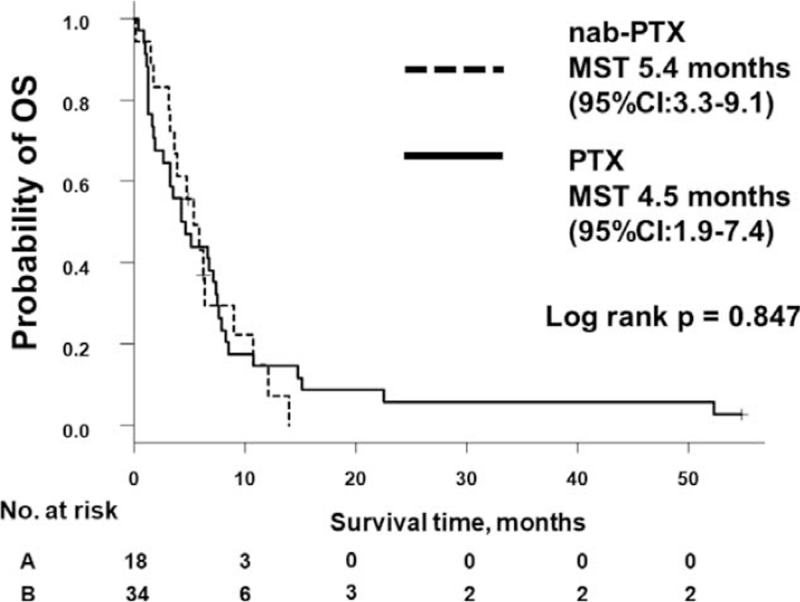
Kaplan-Meier analysis of OS rate. CI = confidence interval, MST = median survival time, nab-PTX = nanoparticle albumin-bound paclitaxel, OS = overall survival, PTX = paclitaxel.

The median number of treatment cycles was 2 (range = 1–8 cycles) with weekly nab-PTX and 1 (range = 1–13 cycles) with weekly PTX. The ORR with nab-PTX and PTX was 5.6% and 8.8%, respectively (*P* = .999), and the DCR was 56% and 38% (*P* = .257) (Table 2). Median PFS was 3.2 (95% confidence interval [CI], 1.8–3.8) months with nab-PTX and 1.7 (95% CI, 1.4–3.4) months with PTX (log-rank *P* = .353) (Fig. 1). Median OS was 5.4 (95% CI, 3.3–9.1) months with nab-PTX and 4.5 (95% CI, 1.9–7.4) months with PTX (log-rank *P* = .847) (Fig. 2). There was no difference in OS or PFS between nab-PTX and PTX.

**Table 2:** Response data.

		No. of patient						
		CR	PR	SD	PD	NE	ORR (%)	DCR (%)
nab-PTX	n = 18	0	1	9	7	1	5.6	56
PTX	n = 34	0	3	10	18	3	8.8	38
*P* value							.999	.257

CR = complete response, DCR = disease control rate, nab-PTX = nanoparticle albumin-bound paclitaxel, NE = not evaluated, ORR = objective response rate, PD = progressive disease, PR = partial response, PTX = paclitaxel, SD = stable disease.

### 3.3 Adverse events

The AE profile is summarized in Table 3. With nab-PTX, Grade 3 or greater AEs were leukopenia (23%), neutropenia (23%), anemia (11%), fatigue (11%), thrombocytopenia (6%), liver dysfunction (6%), pneumonitis (6%), and lung infection (6%). With PTX, Grade 3 or greater AEs were leukopenia (6%), neutropenia (18%), anemia (6%), liver dysfunction (3%), pneumonitis (6%), and lung infection (9%). There was no statistical difference between the 2 groups in Grade 3 or greater events among these AEs. The incidence of any grade neuropathy in the 2 groups was not statistically different (28% in nab-PTX, 35% in PTX, *P* = .758). No acute exacerbation of ILD or treatment-related mortality was observed in either group.

**Table 3:** Toxicity profile.

	No. of patients with event by grade							
	nab-PTX (n = 18)			PTX (n = 34)				
	Any grade	Grade 3	Grade 4	Any grade	Grade 3	Grade 4	*P* value for any grade	*P* value for G3 and G4
Nausea	2 (11%)	0	0	1 (3%)	0	0	.272	–
Vomiting	2 (11%)	0	0	0	0	0	.115	–
Fatigue	17 (94%)	2 (11%)	0	7 (21%)	0	0	<.001	.115
Taste alteration	8 (44%)	0	0	8 (24%)	0	0	.206	–
Neuropathy	5 (28%)	0	0	12 (35%)	0	0	.758	–
Constipation	16 (89%)	0	0	12 (35%)	0	0	<.001	–
Diarrhea	5 (28%)	0	0	6 (18%)	0	0	.482	–
Mucositis	4 (22%)	0	0	3 (9%)	0	0	.218	–
Leukopenia	10 (55%)	3 (17%)	1 (6%)	11 (32%)	1 (3%)	1 (3%)	.141	.166
Neutropenia	11 (61%)	1 (6%)	3 (17%)	12 (35%)	5 (15%)	1 (3%)	.088	.723
Anemia	10 (55%)	2 (11%)	0	17 (50%)	2 (6%)	0	.776	.602
Thrombocytopenia	7 (39%)	0	1 (6%)	4 (12%)	0	0	.034	.346
Liver dysfunction	7 (39%)	0	1 (6%)	11 (32%)	1 (3%)	0	.761	.999
Pneumonitis	1 (6%)	1 (6%)	0	2 (6%)	2 (6%)	0	.999	.999
Lung infection	3 (17%)	1 (6%)	1 (6%)	4 (12%)	3 (9%)	0	.682	.999

nab-PTX = nanoparticle albumin-bound paclitaxel, PTX = paclitaxel.

## 4 Discussion

We examined the efficacy and safety of weekly nab-PTX and weekly PTX for relapsed SCLC. The efficacy of weekly nab-PTX in our study seems to be similar to that of weekly PTX. Of note, in terms of neurotoxicity, weekly nab-PTX showed a favorable profile compared to weekly PTX, consistent with the reported data on nab-PTX in NSCLC.^[5]^

The disadvantage of weekly PTX therapy is seen especially in terms of neurotoxicity. Weekly PTX was reported in 2 prospective phase II studies, which showed ORR of 23% to 29%, and median OS of 3.3 to 5.8 months.^[7,8]^ As a result, weekly PTX is considered to be one of the treatment options. Nab-PTX is expected to have the same treatment potential for relapsed SCLC. Naito et al^[9]^ reported on nab-PTX monotherapy (n = 9) for relapsed SCLC in a retrospective study, and found that ORR, DCR, median PFS, and median OS were 11%, 44%, 2.0 months, and 4.0 months, respectively. Another study that included PTX (n = 27) and nab-PTX (n = 4) regimens reported the ORR, DCR, median PFS, and median OS to be 5%, 58%, 2.2 months, and 4.4 months.^[10]^ Recently, a prospective phase II study of nab-PTX in relapsed SCLC showed 8% ORR, 30% DCR, 1.8 months median PFS, and 3.6 months median OS.^[11]^ In our study, survival with both nab-PTX and PTX was comparable to these previous results.

The AEs in the present study in each group were acceptable in terms of severity. According to 2 previous studies, both of which included ILD, pneumonitis caused by nab-PTX was 0% to 5%.^[9,14]^ In a study of weekly PTX, pneumonitis occurred in 1 of 21 (4.7%) patients, but ILD was excluded from the study cohort.^[8]^ In this study, grade 3 pneumonitis was observed in 6% of patients with both nab-PTX and PTX. Acute exacerbation of ILD was not observed, while 10 of 18 patients in the nab-PTX group and 16 of 34 patients in the PTX group had underlying ILD. These results were consistent with those of previous studies on weekly nab-PTX and weekly PTX for relapsed SCLC.

In previous studies, neuropathy was seen in 3 of 9 patients (all grade 1–2)^[9]^ and 5 of 9 patients (4 grade 1, 1 grade 2) with nab-PTX monotherapy.^[12]^ With weekly PTX regimens, the AE of neuropathy was reported in 12 of 21 patients (10 grade 1 or 2, 2 grade 3)^[8]^ and 18 of 24 patients (all grade 1–2).^[7]^ In the present study, 5 of 18 patients developed neuropathy with the nab-PTX regimen, which were only grades 1–2. Consistent with previous reports, the rate of neurotoxicity profile might be lower with the weekly nab-PTX than with weekly PTX. Collectively, nab-PTX regimens are well tolerated as PTX regimens.

This study has some limitations. First, the dose of nab-PTX, which was 80 mg/m^2^, was lower than in clinical trials of NSCLC (100 mg/m^2^). In this study, the dose of nab-PTX was set to be the same as that of PTX. However, it is unclear whether this dose was more appropriate than 100 mg/m^2^. Thus, in the future it will be necessary to examine whether weekly nab-PTX and weekly PTX are appropriate at a dose of 80 mg/m^2^. Second, this study was a small-sized, single-center, retrospective study. We think that more cases and more data are needed to examine the efficacy of weekly nab-PTX for relapsed SCLC.

In conclusion, the weekly nab-PTX regimen and weekly PTX regimen showed similar activity for relapsed SCLC. In terms of toxicity, the weekly nab-PTX might be less toxic than weekly PTX. Although this is a retrospective single-center study, nab-PTX might be a treatment option alongside PTX for relapsed SCLC.

## Acknowledgments

The authors wish to thank all the study participants and their families.

## Author contributions

**Conceptualization:** Hajime Oi, Toshiaki Matsuda, Tomoki Kimura, Masahiro Morise, Yasuhiko Yamano, Toshiki Yokoyama, Kensuke Kataoka, Yasuhiro Kondoh.

**Data curation:** Hajime Oi, Toshiaki Matsuda, Tomoki Kimura, Masahiro Morise, Yasuhiko Yamano, Toshiki Yokoyama, Kensuke Kataoka, Yasuhiro Kondoh.

**Formal analysis:** Hajime Oi, Toshiaki Matsuda, Tomoki Kimura, Masahiro Morise, Yasuhiko Yamano, Toshiki Yokoyama, Kensuke Kataoka, Yasuhiro Kondoh.

**Funding acquisition:** Hajime Oi, Toshiaki Matsuda, Tomoki Kimura, Masahiro Morise, Yasuhiko Yamano, Toshiki Yokoyama, Kensuke Kataoka, Yasuhiro Kondoh.

**Investigation:** Hajime Oi, Toshiaki Matsuda, Tomoki Kimura, Yasuhiro Kondoh.

**Methodology:** Hajime Oi, Toshiaki Matsuda, Tomoki Kimura, Masahiro Morise, Yasuhiro Kondoh.

**Project administration:** Hajime Oi, Toshiaki Matsuda, Tomoki Kimura, Yasuhiro Kondoh.

**Resources:** Hajime Oi, Toshiaki Matsuda, Tomoki Kimura, Yasuhiro Kondoh.

**Software:** Hajime Oi, Toshiaki Matsuda, Tomoki Kimura, Yasuhiro Kondoh.

**Supervision:** Hajime Oi, Toshiaki Matsuda, Tomoki Kimura, Masahiro Morise, Yasuhiko Yamano, Toshiki Yokoyama, Kensuke Kataoka, Yasuhiro Kondoh.

**Validation:** Hajime Oi, Toshiaki Matsuda, Tomoki Kimura, Masahiro Morise, Yasuhiko Yamano, Toshiki Yokoyama, Kensuke Kataoka, Yasuhiro Kondoh.

**Visualization:** Hajime Oi, Toshiaki Matsuda, Tomoki Kimura, Masahiro Morise, Yasuhiko Yamano, Toshiki Yokoyama, Kensuke Kataoka, Yasuhiro Kondoh.

**Writing – original draft:** Hajime Oi, Toshiaki Matsuda, Tomoki Kimura, Masahiro Morise, Yasuhiro Kondoh.

**Writing – review & editing:** Hajime Oi, Toshiaki Matsuda, Tomoki Kimura, Masahiro Morise, Yasuhiko Yamano, Toshiki Yokoyama, Kensuke Kataoka, Yasuhiro Kondoh.
